# Affinity Maturation of a T-Cell Receptor-Like Antibody Specific for a Cytomegalovirus pp65-Derived Peptide Presented by HLA-A*02:01

**DOI:** 10.3390/ijms22052349

**Published:** 2021-02-26

**Authors:** Se-Young Lee, Deok-Han Ko, Min-Jeong Son, Jeong-Ah Kim, Keunok Jung, Yong-Sung Kim

**Affiliations:** 1 Department of Molecular Science and Technology, Ajou University, Suwon 16499, Korea; sylee1117@ajou.ac.kr (S.-Y.L.); kdh701@ajou.ac.kr (D.-H.K.); minjeong96610@ajou.ac.kr (M.-J.S.); rhwjd319@ajou.ac.kr (J.-A.K.); 2 Department of Allergy and Clinical Immunology, Ajou University School of Medicine, Suwon 16499, Korea; jung2767@ajou.ac.kr

**Keywords:** cytomegalovirus, peptide/major histocompatibility complex class I complex, T-cell-receptor-like antibody, affinity maturation, yeast surface display

## Abstract

Human cytomegalovirus (CMV) infection is widespread among adults (60–90%) and is usually undetected in healthy individuals without symptoms but can cause severe diseases in immunocompromised hosts. T-cell receptor (TCR)-like antibodies (Abs), which recognize complex antigens (peptide–MHC complex, pMHC) composed of MHC molecules with embedded short peptides derived from intracellular proteins, including pathogenic viral proteins, can serve as diagnostic and/or therapeutic agents. In this study, we aimed to engineer a TCR-like Ab specific for pMHC comprising a CMV pp65 protein-derived peptide (^495^NLVPMVATV^503^; hereafter, CMVpp65_495-503_) in complex with MHC-I molecule human leukocyte antigen (HLA)-A*02:01 (CMVpp65_495-503_/HLA-A*02:01) to increase affinity by sequential mutagenesis of complementarity-determining regions using yeast surface display technology. Compared with the parental Ab, the final generated Ab (C1-17) showed ~67-fold enhanced binding affinity (*K_D_* ≈ 5.2 nM) for the soluble pMHC, thereby detecting the cell surface-displayed CMVpp65_495-503_/HLA-A*02:01 complex with high sensitivity and exquisite specificity. Thus, the new high-affinity TCR-like Ab may be used for the detection and treatment of CMV infection.

## 1. Introduction

Human cytomegalovirus (CMV), a β-herpes virus with a double-stranded DNA, infects a wide variety of cells and establishes latency in the host [1]. CMV infection is very common in adults (60‒90% of the population), with higher infection rates with age [2], and is usually asymptomatic in healthy subjects but can cause severe diseases in immunocompromised patients with cellular immunosuppression or immunodeficiency, including transplant recipients and fetuses [1,3].

Major histocompatibility complex class I (MHC-I) molecules, also known as human leukocyte antigen I (HLA-I), are cell-surface antigen-presenting proteins displaying peptide fragments (8–10 amino acid residues in length) derived from intracellular cytoplasmic proteins, including self, viral, and tumor antigens, for recognition by CD8^+^ T cells [4]. In CMV-seropositive hosts, matrix protein pp65 is among the most frequently immunologically recognized CMV antigens [5], accounting for 70–90% of the cytotoxic CD8^+^ T cells’ (CTLs) response to CMV [6]. Among the pp65-derived CTL epitope peptides, the 9-mer peptide ^495^NLVPMVATV^503^ (residues 495–503; hereafter referred to as CMVpp65_495-503_ peptide) is the most immunogenic T cell epitope predominantly displayed on HLA-A*02:01, the most common MHC-I allele in the population [6,7,8]. Hence, detection and targeting of the highly prevalent CMVpp65_495-503_/HLA-A*02:01 complex on the surface of CMV-infected cells are crucial for the development of detection and/or therapeutic modalities [9,10]. T-cell receptors (TCRs) specifically recognize the peptide–MHC complex (pMHC), but their natural affinity is limited to ~1–100 μM [4]. Alternatively, antibodies (Abs) called TCR-like Abs can be engineered to specifically recognize pMHC with high affinity [9,11].

A number of TCR-like Abs directed toward a particular pMHC derived from a pathogenic viral protein or a tumor-associated antigen have been developed because such Abs have many desirable features of conventional immunoglobulin G (IgG) Abs, including large-scale manufacturing capacity and long serum half-life [11]. However, few of these Abs have reached clinical application, and the optimal specificity and affinity of TCR-like Abs need to be defined. High-affinity TCR-like Abs have several potential biomedical applications and may be valuable research reagents for detecting specific virus-/tumor-associated pMHCs on cell and tissue surfaces [11,12].

Previously, a TCR-like Ab (H9) specific for the CMVpp65_495-503_/HLA-A*02:01 complex was reported [13]. However, the affinity was relatively weak (*K_D_* = 300 nM), limiting its potential use as a detection or therapeutic reagent. Here, we aimed to engineer H9 to increase its affinity by ~67-fold for pMHC comprising the CMVpp65_495-503_/HLA-A*02:01 complex by yeast surface display (YSD) technology, thereby enabling highly sensitive and specific detection of the cell surface-displayed pMHC.

## 2. Results

### 2.1. Evaluation of Parental H9

**Figure 1:**
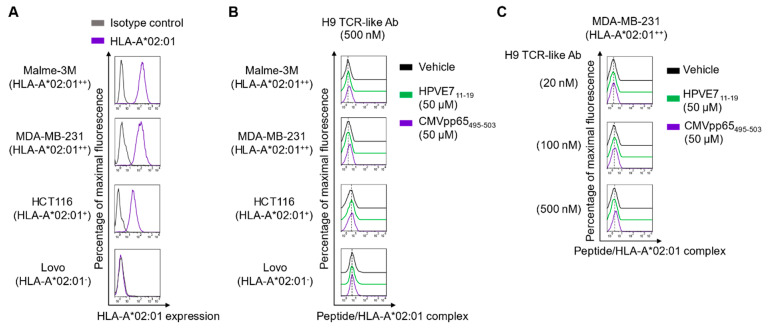
Evaluation of H9 binding to the cell surface-displayed CMVpp65_495-503_/HLA-A*02:01 complex. (**A**) Flow cytometric analysis of the cell surface expression levels of human leukocyte antigen (HLA)-A*02:01, classified as + + (high level) for both MDA-MB-231 and Malme-3M cells, as + (positive) for HCT116 cells, and as ‒ (negative) for LoVo cells. (**B**,**C**) Flow cytometric analysis of H9 binding at 500 nM (B) to peptide-pulsed cells (B) and at various concentrations to peptide-pulsed MDA-MB-231 cells (**C**). Cells were pulsed with the vehicle, CMVpp65_495-503_ peptide (50 μM), or the control HLA-A*02:01-restricted HPVE7_11-19_ (50 μM) peptide for 3 h at 37 °C and incubated with H9 and then the Alexa Fluor 647-conjugated goat anti-mouse immunoglobulin G (IgG)-specific (Fab’)_2_ antibody (Ab) (secondary Ab) prior to flow cytometry. In (A–C), representative histograms from two independent experiments are depicted.

The TCR-like H9 antigen-binding fragment (Fab) was previously isolated by screening a large phage-displayed human Fab library against a recombinant CMVpp65_495-503_/HLA-A*02:01 complex [13]. H9 reformatted into the bivalent IgG form showed binding specificity to soluble pMHC comprising the CMVpp65_495-503_/HLA-A*02:01 complex with relatively weak binding affinity (*K_D_* ≈ 300 nM) [13]. Here, we generated H9 in the mouse IgG2a/κ format and evaluated its binding activity by flow cytometry toward the cell surface-displayed CMVpp65_495-503_/HLA-A*02:01 complex, generated by external peptide pulsing of cells expressing HLA-A*02:01 at various levels (Figure 1A). Even at 500 nM, H9 manifested very weak binding activity only toward MDA-MB-231 and Malme-3M cells expressing HLA-A*02:01 at relatively high levels (HLA-A*02:01^++^) but negligible or little binding activity toward HCT116 cells expressing HLA-A*02:01 at moderate levels (HLA-A*02:01^+^) and toward HLA-A*02:01-negative LoVo cells (Figure 1B). At 100 and 20 nM, H9 binding to peptide-loaded HLA-A*02:01^++^ MDA-MB-231 cells was negligible (Figure 1C). H9 did not react with cells loaded with an off-target peptide of HLA-A*02:01-restricted human papilloma virus (HPV) type 16 E7 protein-derived 9-mer peptide, HPVE7_11-19_ (^11^YMLDLQPETV^19^). These results confirmed the specific binding of H9 to the membrane-bound pMHC comprising the CMVpp65_495-503_/HLA-A*02:01 complex.

However, the binding strength was too weak to detect the complex on cells expressing HLA-A*02:01 at moderate levels. Thus, we sought to engineer H9 for affinity improvement.

### 2.2. Affinity Maturation of H9 to Generate C1 Ab

**Figure 2:**
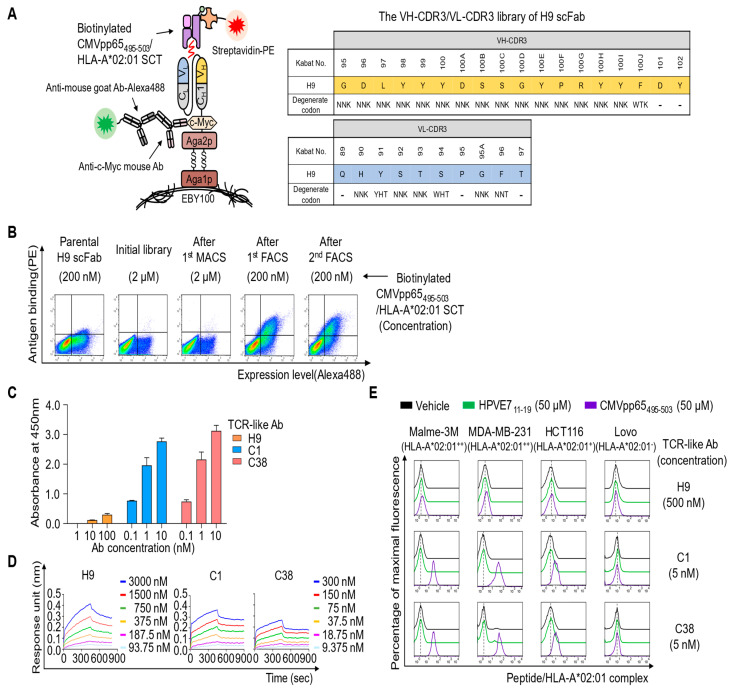
Affinity maturation of H9 and characterization of the isolated clones. (**A**) The scheme of library construction and screening for H9 in the single-chain antigen-binding fragment (scFab) format using YSD technology. The indicated residues in VH-CDR3 and VL-CDR3 were randomized with the indicated degenerate codons. The “‒” sign denotes conserved residues.(**B**) Flow cytometric analysis of antigen binding and expression levels of the yeast surface-displayed scFab library pool enriched after each round of screening by magnetically activated cell sorting (MACS) and fluorescence-activated cell sorting (FACS), compared with those of the parental H9 scFab. (**C**) Dose-dependent binding activity of the isolated and purified Abs in mouse IgG2a/κ form toward the microtiter plate coated with peptide–MHC complex (pMHC) comprising CMVpp65_495-503_/HLA-A*02:01 single-chain trimer (SCT) antigen, as determined by ELISA. (**D**) Binding isotherms of the immobilized Abs toward the soluble CMVpp65_495-503_/HLA-A*02:01 SCT antigen, as measured by biolayer interferometry. pMHC concentrations are indicated (colored). The kinetic interaction parameters are listed in Table 1. (**E**) Flow cytometric analysis of the binding of the isolated T-cell receptor (TCR)-like Abs at the indicated concentrations to the peptide-pulsed cells. Peptide pulsing and flow cytometric analysis were performed as described in Figure 1C. Representative histograms from two independent experiments are depicted.

Owing to lack of information regarding specific amino acid residue interactions between H9 and the CMVpp65_495-503_/HLA-A*02:01 complex, for affinity maturation, we first generated an H9 library by randomization of the third complementarity-determining region (CDR) of variable regions of the heavy chain (VH) and (VL), i.e., VH-CDR3 and VL-CDR3, known to be major contributors to Ab–antigen interaction [14]. Most residues in VH-CDR3 (residues 95–102 in Kabat numbering [15]) and VL-CDR3 (residues 89–97) were randomized with degenerate codons, including the NNK codon encoding all 20 amino acids and one stop codon (Figure 2A). To improve the stability and folding efficiency of the Ab, some highly conserved amino acid residues based on human germline sequences, inferred from the International ImMunoGeneTics information system database [16], were conserved or minimally randomized to maintain the parental amino acid residues at a high frequency. Specifically, in the last three residues of VH-CDR3 (100J, 101, and 102), which are highly conserved with a consensus sequence of 100JPhe/Met/Ile–Asp–Tyr102, only the PheH100J residue was randomized with the degenerate codon WTK (encoding F, I, M, and L) while preserving the other residues, AspH101 and TyrH102. Similarly, for VL-CDR3, the highly conserved residues GlnL89, ProL95, and ThrL97 were retained owing to their high frequency in the human germline sequences. Residues TyrL91, SerL94, and PheL96 were mutated with degenerate codons YHT (encoding F, S, Y, L, P, and H), WHT (encoding F, S, Y, I, T, and N), NNT (encoding F, S, Y, C, L, P, H, R, I, T, N, S, V, A, D, and G), respectively (Figure 2A). The VH-CDR3/VL-CDR3-randomized H9 library was generated by YSD technology in the single-chain Fab (scFab) format, wherein the C-terminus of VL was linked to the N-terminus of VH via a G4S-based 63-amino-acid linker (Figure 2A) [17,18]. The library diversity was ~1.5 × 107, and sequencing of tens of clones confirmed the fidelity of the library diversity.

For library screening, we prepared the soluble antigen of CMVpp65_495-503_/HLA-A*02:01 single-chain trimer (SCT) protein with a C-terminal Avi tag (for biotinylation) (Supplementary Figure S1). We engineered the SCT form to have an artificial disulfide bridge between the HLA α1 domain (Tyr108Cys) and linker L1 (position 2 of L1) to maintain stable binding of CMVpp65_495-503_ into the groove of the MHC-I complex (Supplementary Figure S1) [19,20]. The disulfide-bonded SCT format ensured that the TCR-like Ab does not recognize MHC-I alone. As an off-target antigen, the HPVE7_11-19_/HLA-A*02:01 SCT protein was prepared similarly. The pMHC SCT proteins were expressed in cultured HEK293F cells. The purified protein (~49.7 kDa) was site-specifically biotinylated, as confirmed by a streptavidin gel shift assay (Supplementary Figure S1C).

The H9 library was screened by one round of magnetically activated cell sorting (MACS), followed by two rounds of fluorescence-activated cell sorting (FACS) with the biotinylated CMVpp65_495-503_/HLA-A*02:01 SCT antigen in the presence of a 10-fold higher concentration of the non-biotinylated off-target HPVE7_11-19_/HLA-A*02:01 SCT antigen (Figure 2B), thereby yielding two unique good-affinity binders, C1 and C38 scFabs (Supplementary Figure S2). The isolated scFab clones were converted into the mouse IgG2a/κ form and expressed in HEK293F cells. ELISA revealed that purified C1 and C38 bound to the soluble CMVpp65_495-503_/HLA-A*02:01 SCT in proportion to the concentration, thus showing much stronger binding activity than parental H9 (Figure 2C). In a kinetic binding analysis performed by biolayer interferometry, Abs C1 and C38 manifested more than 10-fold stronger affinity (*K*_D_ ≈ 13 and 31 nM, respectively) than that of parental H9 (*K*_D_ ≈ 348 nM; Figure 2D and Table 1). The binding specificity of the affinity-matured TCR-like Abs to the cell surface-displayed CMVpp65_495-503_/HLA-A*02:01 complex was evaluated by flow cytometry using cells pulsed with peptides. Compared with parental H9 at 500 nM, both C1 and C38, even at a 100-fold lower concentration (at 5 nM), exhibited a substantial binding activity toward HLA-A*02:01-positive cells, including HLA-A*02:01^+^ HCT116 cells (Figure 2E). However, the affinity-matured Abs did not bind at all to the same HLA-A*02:01-positive cells loaded with the off-target HPVE7_11-19_ peptide or to HLA-A*02:01-negative LoVo cells (Figure 2E), thereby confirming their binding specificity to the cell surface-displayed CMVpp65_495-503_/HLA-A*02:01 complex. Thus, both Abs C1 and C38 may exhibit improved affinity while maintaining their specificity. 

The association rate constant (*k*_on_), dissociation rate constant (*k*_off_), and equilibrium dissociation constant (*K*_D_) and an estimate of the goodness of curve fit (*R*^2^) were calculated in the Octet Data Analysis software, v.11.0 (ForteBio).

### 2.3. Affinity Maturation of C1 to Generate High-Affinity TCR-Like Abs

**Figure 3:**
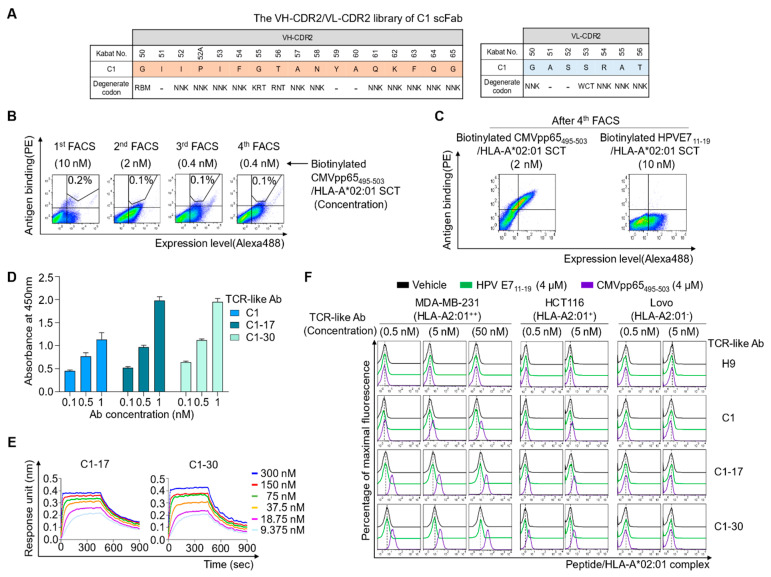
Affinity maturation of C1 Ab to generate high-affinity TCR-like Abs. (**A**) The scheme of yeast scFab library construction for VH-CDR2 and VL-CDR2 of C1 Ab, wherein the indicated residues were randomized with the indicated degenerate codons. The “‒” sign indicates conserved residues. (**B**) Flow cytometric sorting gate plots of the yeast surface-displayed scFab library screening in each round of screening by FACS with the indicated concentration of the biotinylated CMVpp65_495-503_/HLA-A*02:01 SCT antigen in the presence of a 10-fold higher concentration of the non-biotinylated off-target HPVE7_11-19_/HLA-A*02:01 SCT protein. (**C**) Flow cytometric analysis of target-specific enrichment for the yeast surface-displayed scFab library pool enriched after four rounds of FACS using the indicated target and off-target antigen. (**D**) Dose-dependent binding activity of the isolated and purified Abs in mouse IgG2a/κ form toward the microtiter plate coated with pMHC comprising CMVpp65_495-503_/HLA-A*02:01 complex antigen, as determined by ELISA. (**E**) Binding isotherms of the immobilized IgG2a/κ Abs toward the soluble CMVpp65_495-503_/HLA-A*02:01 complex antigen, as measured by biolayer interferometry. The concentrations of pMHC are indicated (colored). The kinetic interaction parameters are listed in Table 1. (**F**) Flow cytometric analysis of binding of the TCR-like Abs in IgG2a/κ form at the indicated concentrations to peptide-pulsed cells. Cells were pulsed for 3 h at 37 °C with the vehicle, CMVpp65_495-503_ peptide (4 μM), or the control, HLA-A*02:01-restricted HPVE7_11-19_ (4 μM) peptide, and incubated with the TCR-like Abs at the indicated concentrations and then with the Alexa Fluor 647-conugated goat anti-mouse IgG-specific (Fab’)_2_ Ab (secondary Ab) prior to flow cytometry. Representative histograms from two independent experiments are depicted.

Considering the very low density of specific peptide/HLA complexes on a natural cell surface (≤1000 per cell [21]), successful therapeutic and detection use of a TCR-like Ab requires strong affinity and high specificity [22]. Therefore, we selected C1, which has higher affinity than C38, for the next round of affinity maturation. For affinity maturation of C1, the VH-CDR2 (residues 50–65) and VL-CDR2 (residues 50–56) regions (except for the residues generally conserved in human germline sequences, e.g., IleH51, TyrH59, and AlaH60 in VH-CDR2 and AlaL51 and SerL52 in VL-CDR2) were randomized using degenerate codons (Figure 3A). The library was generated in the scFab format by YSD technology with a diversity of ~1.3 × 10^7^ and was screened by four rounds of FACS against the biotinylated antigen, CMVpp65_495-503_/HLA-A*02:01 SCT, with a gradual decrease in antigen concentration in the presence of a 10-fold higher concentration of the non-biotinylated off-target competitor, HPVE7_11-19_/HLA-A*02:01 SCT (Figure 3B,C). Analysis of more than 50 finally isolated clones yielded two unique clones, C1-17 and C1-30 (Supplementary Figure S2). The isolated clones were reformatted into mouse IgG2a/κ form and purified for further characterization. ELISA indicated improved binding activity of both C1-17 and C1-30 for the soluble CMVpp65_495-503_/HLA-A*02:01 SCT compared with C1 (Figure 3D). Binding kinetics analysis revealed that C1-17 and C1-30 showed single-digit nanomolar affinities (*K*_D_) of ~5.2 and ~8.7 nM, respectively, which were approximately twofold stronger than that of parental C1 (*K*_D_ ≈ 13 nM; Table 1). In all cases, affinity improvement was essentially owing to an increase in the association rate constant *k*_on_ (Figure 3E and Table 1).

Next, we assessed the specificity and lower detection limits of the affinity-matured Abs toward cells pulsed with the peptide at a low concentration (down to 4 μM) to generate low-density CMVpp65_495-503_/HLA-A*02:01 complex on cells. Both C1-17 and C1-30 strongly stained HLA-A*02:01-positive cells loaded with CMVpp65_495-503_ in proportion to the concentration but did not stain the same cells pulsed with a vehicle or the off-target HPVE7_11-19_ peptide and HLA-A*02:01-negative cells (Figure 3F). Although parental C1 at a low concentration of 0.5 nM failed to detect the membrane-bound CMVpp65_495-503_/HLA-A*02:01 complex, both C1-17 and C1-30 at the same concentration detected it (Figure 3F). Thus, affinity-matured TCR-like Abs C1-17 and C1-30 reliably detected the cell surface-displayed CMVpp65_495-503_/HLA-A*02:01 complex with high sensitivity and exquisite specificity.

## 3. Discussion

The low affinity of TCR-like Abs is one of the major hurdles associated with their detection and therapeutic applications. We engineered a TCR-like Ab H9 specific for the CMVpp65_495-503_/HLA-A*02:01 complex to improve H9′s affinity while retaining its specificity. We performed two rounds of affinity maturation by sequential random mutagenesis on the VH-/VL-CDR3 of H9 and then on the VH-/VL-CDR2 of C1 in the scFab format using YSD technology. The finally generated, highest-affinity Ab C1-17 possessed ~67-fold improved affinity (*K*_D_ ≈ 5.2 nM) compared with that of the parental H9 (*K*_D_ ≈ 348 nM). Parental H9 failed to detect the membrane-bound CMVpp65_495-503_/HLA-A*02:01 complex even on HLA-A*02:01^++^ cells at a concentration below 500 nM. Conversely, both C1-17 and C1-30 with single-digit nanomolar affinities detected the cell surface-displayed CMVpp65_495-503_/HLA-A*02:01 complex at a 1000-fold lower concentration (0.5 nM) even on HLA-A*02:01^+^ HCT116 cells, thereby showing high sensitivity owing to affinity maturation. They did not bind to HLA-A*02:01-positive cells, unpulsed or pulsed with an off-target peptide, nor to HLA-A*02:01-negative cells, thus confirming their exquisite specificity.

The expression levels of the pMHC complex on the cell surface are relatively low, ranging from tens to hundreds of molecules/cell, compared with other membrane receptors [11]. For example, the CMVpp65_495-503_/HLA-A*02:01 complex was reported to be ~100 molecules/cell on the surface of CMV-infected fibroblasts [13]. Accordingly, TCRs or TCR-like Abs with high affinity and specificity are necessary for the sensitive detection or targeting of the low copy numbers of pMHC [10,11,22]. Though the H9 Ab detected the CMVpp65_495-503_/HLA-A*02:01 on the surface of CMV-infected fibroblasts [13], it has not been further developed. The high-affinity TCR-like C1-17 Ab, engineered to have a *K*_D_ ≈ 5.2 nM in this study, can be developed as a research agent to detect CMVpp65_495-503_/HLA-A*02:01 presentation on the surface of and inside cells during CMV infection and as a therapeutic agent to eliminate CMV-infected cells.

The full-length TCR-like Ab was generated based on the Fc portion of mouse IgG2a rather than that of human IgG isotype for use as a detection agent for the CMVpp65_495-503_/HLA-A*02:01 complex on human cells and tissues. The mouse IgG2a isotype also has merits as a primary Ab in detection because it exhibits a detection sensitivity with labeled anti-mouse IgG isotype-specific secondary Abs that is superior to that of the other mouse IgG isotype Abs [23], and the anti-mouse IgG2a-specific secondary Abs are readily available. To be used as a therapeutic Ab, the constant regions of the TCR-like Ab need to be switched into human IgG1 with greater effector functions than the other isotypes [24].

A few antiviral drugs, including ganciclovir and valganciclovir, have been used for treating CMV infection, but viral resistance is a major challenge associated with their use [1]. Another approach is the transfer of donor-derived CMV-specific CTLs, but it remains limited due to the occurrence of graft-versus-host disease (GVHD) in allogeneic recipients [25]. The high-affinity TCR-like Ab C1-17 can be converted into a bispecific T-cell engager [20] and a chimeric antigen receptor (CAR) for CAR-T therapy based on the autologous T-cells to overcome allogeneic immunogenicity [3,26]. Moreover, C1-17 can be developed as a therapeutic Ab to eliminate CMV-infected cells through the effecter functions, such as Ab-dependent cellular cytotoxicity [27,28], or via a targeting agent to deliver cytotoxic payloads, such as potent drugs and toxins [9,29]. Comparative analyses of CTL responses in CMV-seropositive individuals have shown that, among the CMV-derived CTL epitopes, the pp65-derived CMVpp65_495-503_ and the major immediate-early gene product (IE-1)-derived VLEETSVML peptide (residues 316–324) are the most frequent CTL epitope peptides, with the former being more dominant than the latter [5,6,30]. CMVpp65_495-503_ is predominantly presented by HLA-A*02:01, one of the most frequent MHC-I alleles in the human population (30~50%, depending on the ethnicity) [27]. Accordingly, C1-17 specific for the CMVpp65_495-503_/HLA-A*02:01 complex could be used in a maximum of up to ~50% of CMV-infected individuals as a detection or therapeutic agent. Nonetheless, C1-17, restricted to the single HLA-A*02:01 allele, is not suitable for broad applicability due to the three HLA genes and their thousands of polymorphic alleles in humans [9].

This study has some limitations. The engineered TCR-like Abs specific for CMVpp65_495-503_-bound HLA-A*02:01 were evaluated only for cells exogenously pulsed with peptides. Thus, the high-affinity TCR-like C1-17 Ab must be further validated as a potential detection and/or therapeutic agent for the pMHC naturally presented on CMV-infected cells, such as fibroblasts, epithelial cells, endothelial cells, neurons, monocytes, and macrophages, which are susceptible to CMV infection [31,32,33], in comparison with the lower-affinity clones, including the parent H9 Ab [13].

In conclusion, we developed a high-affinity TCR-like Ab (C1-17) specific for the highly prevalent pMHC of CMV infection, i.e., the CMVpp65_495-503_/HLA-A*02:01 complex, in both soluble and membrane-bound forms. In addition to its value as a study reagent, the high-affinity TCR-like Ab can be utilized as a therapeutic agent against CMV infection.

## 4. Materials and Methods

### 4.1. Peptides and Plasmids

Human CMV pp65-derived 9-mer peptide, CMVpp65_495-503_ (^495^NLVPMVATV^503^), and human papilloma virus (HPV) type 16 E7 protein-derived 9-mer peptide, HPVE7_11-19_ (^11^YMLDLQPETV^19^), were synthesized with 95% purity (AnyGen, Gwangju, Korea). DNA fragments encoding the variable regions of the heavy chain (VH) and light chain (VL) of H9 (patent US8361473B2) were synthesized (Bioneer, Daejeon, Korea), and respective VH and VL genes were subcloned into a modified pcDNA 3.4 VH vector (Invitrogen, CA, USA) carrying the mouse IgG2a constant domain and a pcDNA 3.4 VL vector carrying the mouse kappa constant domain, respectively [34,35], to be expressed in mouse IgG2a/κ form. Similarly, engineered H9-derived Abs were subcloned. DNA encoding the full-length HLA-A*02:01 (residues 25–298, GenBank accession #: BC019236) was purchased from SinoBiological (cat. # HG13263-CH, Korea), and the human β2-microglobulin (β2m) gene was prepared by DNA synthesis (Bioneer, Daejeon, Korea). To express the recombinant pMHC protein in the single-chain trimer (SCT) form [19,20], the open-reading frame of the target (CMVpp65_495–503_) or off-target (HPVE7_11–19_) peptide‒GCGGS(G_4_S)_2_ linker‒β2m‒(G_4_S)_4_ linker‒extracellular domain of the HLA-A*02:01 protein (residues 25–298) with Y108C mutation‒GS‒Avi tag(GLNDIFEAQKIEWHE)‒GS‒8×His tag was subcloned in-frame downstream of a secretion signal peptide in the pcDNA3.4 vector to be expressed as the CMVpp65_495–503_/HLA-A*02:01 or HPVE7_11–19_/HLA-A*02:01 SCT protein (Supplementary Figure S1).

### 4.2. Expression and Purification of Abs and Proteins

Plasmids encoding the heavy chain and light chain of Abs were transiently co-transfected in pairs, at equivalent molar ratios, into cultured mammalian human embryonic kidney HEK293F cells in Freestyle 293F medium (Invitrogen, CA, USA, 12338018) following the standard protocol [34,35]. Culture supernatants were collected after 6 days by centrifugation and filtration (0.22 μm, polyethersulfone; Corning). Abs were purified from the culture supernatants using a CaptivA™ Protein A-agarose chromatographic column (Repligen, MA, USA) and were extensively dialyzed to achieve the final composition of phosphate-buffered saline (PBS; pH 7.4). Likewise, the plasmid encoding the pMHC SCT protein was transfected into HEK293F cells. The pMHC protein was purified from the culture supernatant using Ni-NTA resin (GE Healthcare, IL, USA). Protein concentrations were determined using a bicinchoninic acid kit (Thermo Fisher Scientific, Waltham, MA, USA). To prepare an Ab-screening antigen, the purified pMHC SCT proteins were biotinylated using a BirA500 kit (Avidity LLC, Colorado, USA) following the manufacturer’s instructions [35].

### 4.3. Enzyme-Linked Immunosorbent Assay (ELISA)

Binding activity and specificity of Abs to the purified CMVpp65_495-503_/HLA-A*02:01 SCT protein were determined by ELISA, as described previously [17].

### 4.4. Cell Cultures

HLA-A*02:01-expressing cell lines Malme-3M, MDA-MB-231, and HCT116 and an HLA-A*02:01-negative LoVo cell line were purchased from the Korean Cell Line Bank and maintained and cultured in an RPMI-1640 medium (HyClone, Busan, Korea) supplemented with 10% heat-inactivated fetal bovine serum (FBS) (HyClone, Busan, Korea), penicillin (100 U/mL), streptomycin (100 μg/mL), and amphotericin B (0.25 μg/mL; HyClone) [35,36]. All cell lines were maintained at 37 °C in a humidified 5% CO_2_ incubator and routinely screened for *Mycoplasma* contamination (CellSafe, Yongin-si, Korea).

### 4.5. Flow Cytometry

To determine the expression levels of HLA-A*02:01, cells (2.0 × 10^5^ cells/mL) were incubated for 30 min with a PE-conjugated mouse anti-HLA-A2 monoclonal Ab (cat. # sc-32236 PE, Santa Cruz Biotechnology, diluted 1:100). After washing with 1 mL ice-cold PBS, cells were analyzed on a FACSCalibur flow cytometer (Becton-Dickinson, Franklin lakes, New Jersey, USA). All staining procedures were performed at 4°C.

To detect pMHC on cell surfaces, cells (3.0 × 10^5^ cells/mL) were pulsed with the vehicle, CMVpp65_495-503_, or HPVE7_11-19_ peptide at the indicated concentration for 3 h at 37 °C, washed with fluorescence-activated cell sorting (FACS) buffer (1% FBS in PBS, pH 7.4), and resuspended at 1.5 × 10^5^ cells/sample. All staining procedures were performed at 4 °C. Cells were incubated for 1 h with the TCR-like Ab at the indicated concentration, washed with 1 mL FACS buffer, and incubated with an Alexa Fluor 647-conjugated goat anti-mouse IgG-specific F(ab’)_2_ polyclonal Ab (cat. # 115-606-008, Jackson ImmunoResearch, diluted 1:600) for 30 min. After washing with 1 mL ice-cold PBS, cells were analyzed on the FACSCalibur flow cytometer. Data were analyzed using FlowJo V10 software (Tree Star).

### 4.6. Affinity Maturation of Abs

The yeast strains and media compositions have been previously described in detail [34,35]. Library generation of Abs by complementarity-determining region (CDR) mutagenesis was performed in the scFab format involving a G_4_S-based 63-amino-acid linker between VL and VH, using YSD technology as described previously [17]. The yeast library was screened using magnetically activated cell sorting (MACS) and an FACS Aria III instrument (BD Biosciences) against biotinylated CMVpp65_495-503_/HLA-A*02:01 SCT protein (with a gradual decrease in concentration from 2 μM to 0.4 nM) in the presence of a 10-fold higher concentration of non-biotinylated HPVE7_11-19_/HLA-A*02:01 SCT protein as a competitor, as specified in the text. In FACS, cell surface expression and antigen binding levels of the scFab library were monitored by indirect double immunofluorescence labeling of the CH1 C-terminal c-myc tag (anti-c-myc mouse Ab [9E10], diluted 1:100) with an Alexa 488-labeled goat anti-mouse IgG Ab (Invitrogen, diluted 1:600) and streptavidin-conjugated R-phycoerythrin (Invitrogen, diluted 1:600). Typically, the top 0.1–0.2% of target-binding cells were sorted. The final sorted yeast cells were plated on a selective medium, and individual clones were isolated and further analyzed. DNA from the screened yeast cells was recovered using a Zymoprep kit (Zymo Research, CA, USA) as previously described [34,35].

### 4.7. Biolayer Interferometry

Kinetic binding interactions of TCR-like Abs with CMVpp65_495-503_/HLA-A*02:01 SCT protein were monitored at pH 7.4 using an Octet QKe System (ForteBio, California, USA), as described previously [17,35]. All data were globally fitted via the 1:1 Langmuir binding model, and association and dissociation rate constants were calculated using Octet Data Analysis Software, version 11.0 (ForteBio, Fremont, CA, USA).

## 5. Patents

Patents resulting from the work reported in this manuscript have been filed in the Republic of Korea (Application number: KR 10-2020-0138273) and PCT (application number: PCT/KR2020/017067).

## Figures and Tables

**Table 1:** Parameters of binding kinetics of TCR-like Abs in relation to the CMVpp65_495-503_/HLA-A*02:01 SCT protein, as measured using biolayer interferometry.

Abs	*K*_D_ (nM)	*k*_on_ (M^−1^s^−1^)	*k*_off_ (s^−1^)	*R* ^2^
H9	348 ± 33	(6.3 ± 3.3) × 10^3^	(2.2 ± 0.2) × 10^−3^	0.97
C1	12.6 ± 0.3	(1.8 ± 0.2) × 10^5^	(2.3 ± 0.4) × 10^−3^	0.97
C38	30.6 ± 0.1	(1.5 ± 0.1) × 10^5^	(4.7 ± 0.4) × 10^−3^	0.98
C1-17	5.2 ± 0.1	(9.3 ± 0.2) × 10^5^	(4.8 ± 0.1) × 10^−3^	0.99
C1-30	8.7 ± 0.1	(8.0 ± 0.2) × 10^5^	(7.0 ± 0.1) × 10^−3^	0.99

## Data Availability

All data in this study are available within the article or from the authors on request.

## Supplementary Materials

The following are available online at https://www.mdpi.com/1422-0067/22/5/2349/s1.
