# Supplementary material for: Affinity Maturation of a T-Cell Receptor-Like Antibody Specific for a Cytomegalovirus pp65-Derived Peptide Presented by HLA-A*02:01
Source: Int J Mol Sci. 2021 Feb 26;22(5):2349. doi: 10.3390/ijms22052349 (PMC7956451; doi:10.3390/ijms22052349)
Supplement: Supplementary file 1 [file ijms-22-02349-s001.pdf]

Supplementary Material

Affinity maturation of a T-cell-receptor-like antibody specific for a cytomegalovirus pp65-derived peptide presented by HLA-A*02:01

Se-Young Lee, Deok-Han Ko, Min-Jeong Son, Jeong-Ah Kim, Keunok Jung, and Yong-Sung Kim

Inventory of Supplementary Information

Supplementary Figure 1
Supplementary Figure 2

Supplementary Figure 1

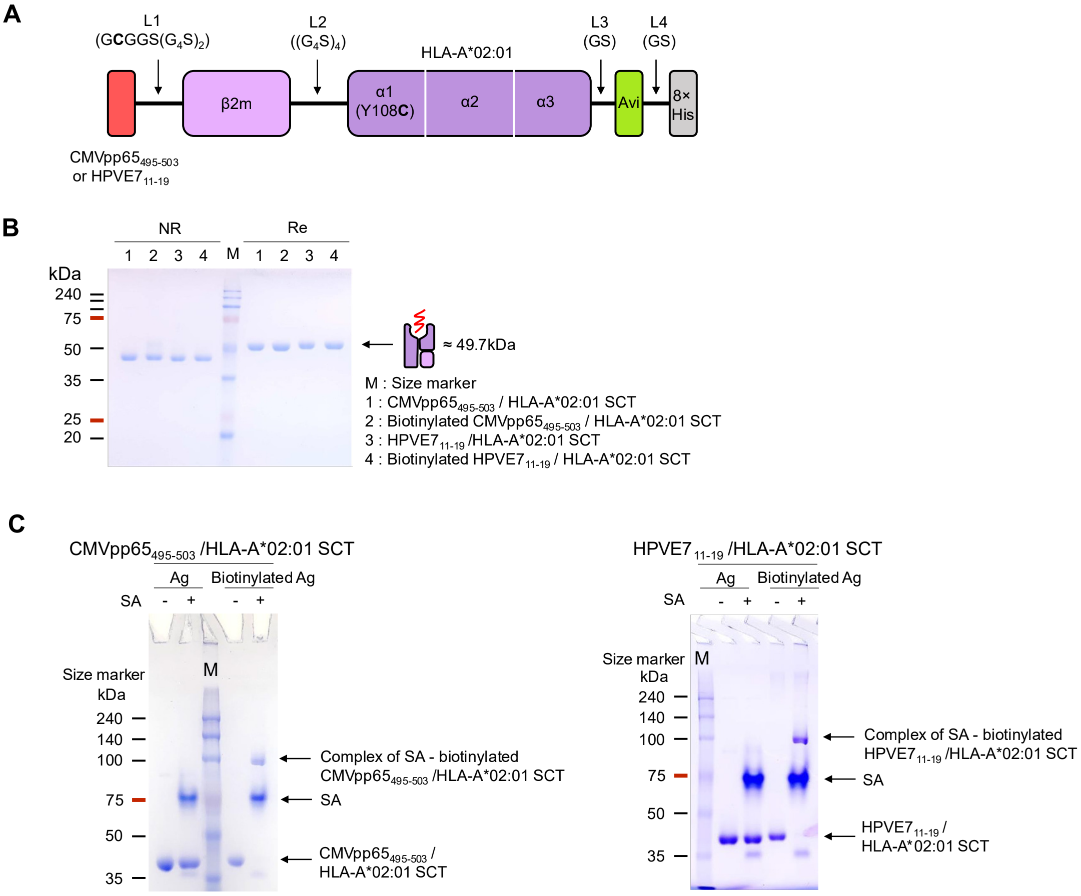

Supplementary Figure S1. Preparation of the target and off-target pMHC proteins.

(**A**) The expression scheme for the CMVpp65$_{495-503}$/HLA-A*02:01 SCT protein. An artificial disulfide bridge was introduced between the HLA α1 domain (Tyr108Cys) and linker L1 (position 2 of L1) to maintain stable binding of the CMVpp65$_{495-503}$ into the groove of the MHC-I complex.

(**B**) The purified CMVpp65$_{495-503}$/HLA-A*02:01 and HPVE7$_{11-19}$/HLA-A*02:01 SCT proteins (biotinylated or nonbiotinylated, 3 µg each) were analyzed by 12% SDS-PAGE under reducing ("Re") or nonreducing ("NR") conditions and then stained with Coomassie Brilliant Blue.

(**C**) Detection of biotinylated SCT proteins by streptavidin (SA)-induced band-shift analysis using SDS-PAGE under reducing conditions. Biotinylation of SCT proteins with the Avi tag (2 mg) was performed using a BirA500 kit (Avidity) following the manufacturer's instructions.

Then, the biotinylated SCT proteins (3 μg, 20 mol) were incubated with SA (80 mol) for 30 min at room temperature and subjected to 8% SDS-PAGE followed by Coomassie Brilliant Blue staining to determine biotinylation extent. Compared with nonbiotinylated SCT proteins, biotinylated SCT proteins in complex with SA featured a dramatic change in migration, thereby confirming biotinylation. The positions of the biotinylated or nonbiotinylated SCT proteins, SA, and the complex of a biotinylated SCT with SA are indicated with arrows.

Supplementary Figure 2

		[VH-CDR1]	[VH-CDR2]	[VH-CDR3]
H9	VH	SYAISW	GIIPIFGTANYAQKFQG	GDLYYYDSSGYPRYYFDY
C1	VH	SYAISW	GIIPIFGTANYAQKFQG	GDLYYYDSSGYP**LW**Y**M**DY
C38	VH	SYAISW	GIIPIFGTANYAQKFQG	GDLYYYDSSGYP**W**YY**M**DY
C1-17	VH	SYAISW	**S**IIPIFG**V**A**E**YA**H**KFQG	GDLYYYDSSGYPLWYMDY
C1-30	VH	SYAISW	**S**IIPIFG**A**A**E**YAQKFQG	GDLYYYDSSGYPLWYMDY

		[VL-CDR1]	[VL-CDR2]	[VL-CDR3]
H9	VL	RASQSVSSSYLA	GASSRAT	QHYSTSPGFT
C1	VL	RASQSVSSSYLA	GASSRAT	Q**D**YST**Y**P**A**FT
C38	VL	RASQSVSSSYLA	GASSRAT	QH**SYAF**PGFT
C1-17	VL	RASQSVSSSYLA	GAS**T**R**P**T	Q**D**YST**Y**P**A**FT
C1-30	VL	RASQSVSSSYLA	GASSR**PR**	Q**D**YST**Y**P**A**FT

Supplementary Figure S2. Amino acid sequence alignment of the isolated clones from the first and second round of affinity maturation focusing on VH-/VL-CDRs.

The mutated residues are highlighted in bold. Clones C1 and C38 were isolated from the VH-CDR3 and VL-CDR3 library of H9 scFab, as presented in Fig. 2A. Clones C1-17 and C1-30 were isolated from the VH-CDR2 and VL-CDR2 library of C1 scFab, as shown in Fig. 3A.
